# The Effects of Acute Stress-Induced Sleep Disturbance on Acoustic Trauma-Induced Tinnitus in Rats

**DOI:** 10.1155/2014/724195

**Published:** 2014-08-03

**Authors:** Yiwen Zheng, Lucy Stiles, Yi-Ting Chien, Cynthia L. Darlington, Paul F. Smith

**Affiliations:** ^1^Department of Pharmacology and Toxicology, School of Medical Sciences, University of Otago, P.O. Box 913, Dunedin 9016, New Zealand; ^2^Brain Health Research Centre, University of Otago, Dunedin 9016, New Zealand

## Abstract

Chronic tinnitus is a debilitating condition and often accompanied by anxiety, depression, and sleep disturbance. It has been suggested that sleep disturbance, such as insomnia, may be a risk factor/predictor for tinnitus-related distress and the two conditions may share common neurobiological mechanisms. This study investigated whether acute stress-induced sleep disturbance could increase the susceptibility to acoustic trauma-induced tinnitus in rats. The animals were exposed to unilateral acoustic trauma 24 h before sleep disturbance being induced using the cage exchange method. Tinnitus perception was assessed behaviourally using a conditioned lick suppression paradigm 3 weeks after the acoustic trauma. Changes in the orexin system in the hypothalamus, which plays an important role in maintaining long-lasting arousal, were also examined using immunohistochemistry. Cage exchange resulted in a significant reduction in the number of sleep episodes and acoustic trauma-induced tinnitus with acoustic features similar to a 32 kHz tone at 100 dB. However, sleep disturbance did not exacerbate the perception of tinnitus in rats. Neither tinnitus alone nor tinnitus plus sleep disturbance altered the number of orexin-expressing neurons. The results suggest that acute sleep disturbance does not cause long-term changes in the number of orexin neurons and does not change the perception of tinnitus induced by acoustic trauma in rats.

## 1. Introduction

Chronic tinnitus (“ringing in the ears”) is a debilitating condition affecting about 10% of the adult population [1]. As ageing occurs, its prevalence increases, affecting 14.3% of the population between the ages of 60 and 69 [1]. Despite numerous studies investigating the underlying mechanisms and therapeutic options, to date, there is no effective treatment for tinnitus. Tinnitus treatment is complicated not only by a poor understanding of its mechanisms but also by the variations in individuals' reactions to its perception. For example, while some people can tolerate their tinnitus, others feel severely distressed and handicapped [2–5]. It has been shown that patients with tinnitus that causes distress often have high rates of psychopathological conditions (see [6–8] for reviews). Specifically, tinnitus severity has been associated with high levels of anxiety, depression, and sleeping disturbance [9–14].

Among tinnitus-related problems, sleeping disturbance is the second most frequent comorbid condition [12], affecting 50–77% of tinnitus patients [15, 16]. Both the subjective (i.e., self-rated) and objective (i.e., electroencephalography (EEG), electromyography (EMG), and electrooculography (EOG) recordings) sleep measurements in tinnitus patients with disturbed sleep are very similar to those that occur in insomnia [14], a sleep disorder characterised by difficulties in initiating/maintaining sleep. It is tempting to think that insomnia is a consequence of people's reaction to the annoyance of tinnitus; however, studies have suggested that insomnia may, in fact, be a risk factor/predictor for tinnitus-related distress [17, 18] and the two conditions may share common neurobiological mechanisms (see [19] for a review).

Recent research has suggested that hyperarousal might play an important role in the pathophysiology of both insomnia and the conscious perception of tinnitus (see [19–22] for reviews). It is assumed that insomnia and tinnitus perception are the result of active cognitive appraisal behaviour that are reinforced by a vicious cycle of biased attention and negative behaviour. While stress-reactivity is the most important risk factor in the development of insomnia, stressful changes in one's life, such as divorce, accidents, or sickness in family members, have been linked to the development of tinnitus and stress is especially important for tinnitus to transit from mild to severe (see [23, 24] for reviews). Therefore, there might be a neurobiological system that regulates the normal arousal level under physiological conditions and a malfunction of this system under stressful conditions could serve as a promoter for hyperarousal in pathological conditions, such as tinnitus and insomnia.

One candidate system is the orexin system located in the lateral hypothalamus. Discovered in 1998, the orexin system is well recognised for its role in sleep-wake regulation, appetite control, reward, emotional responses to stress, and learning and memory (see [25] for a review). There are two peptides (orexin-A and -B) and their corresponding receptors are distributed widely in brain areas involved in arousal, sensory processing, and autonomic function, including key structures involved in processing auditory information, such as the inferior colliculus and the ventral cochlear nucleus [26]. There is evidence that orexin neurons are capable of undergoing experience-induced synaptic plasticity, which is thought to be responsible for the maintenance of long-lasting arousal (see [27] for a review). Inappropriate activation of the orexin system has been attributed to the pathophysiology of insomnia based on the beneficial effects of orexin receptor antagonists [28, 29]. However, the possibility that inappropriate activation of the orexin system may also reset the arousal threshold, to promote tinnitus, has never been explored.

Sleep patterns across many different species have been characterised and rodents, such as rats and mice, normally spend most of their time asleep during the light phase and awake during the dark phase. Compared with humans, sleep in rodents is broken into small segments, with the duration of each sleep episode being only 10–14 min in rats (see [30, 31] for reviews). The different rodent models of insomnia, such as the stress-induced model, caffeine-induced model, genetic model, and brain lesion model, have been used to resemble some aspects of the sleep disturbance that insomnia patients experience; however, none of the models can convincingly represent the human insomniac condition (see [31] for a review). Among these models, the stress-induced insomnia model using cage exchange has been shown to result in sleep disturbance similar to that of patients with stress-induced insomnia [32]. Given that stress is one of the risk factors associated with both tinnitus and sleep disorders (see [23, 33, 34] for reviews), this model is ideal for studying the relationship between stress, sleep disturbance, and tinnitus. Therefore, in this study, we used the stress-inducing cage exchange method to induce sleep disturbance in rats and investigated the susceptibility of these rats to acoustic trauma-induced tinnitus. Furthermore, the number of orexin-expressing neurons in the hypothalamus was also assessed using immunohistochemistry.

## 2. Methods

### 2.1. Animals

Thirty-two male Wistar rats (300–350 g, 2 months old, at the beginning of the experiment) were obtained from the Hercus-Taieri Resource Unit, Dunedin, New Zealand, and were divided into 3 groups (*n* = 12 per group): (1) control (no cage exchange and no acoustic trauma); (2) acoustic trauma + clean cage; and (3) acoustic trauma + dirty cage. The animals were maintained on a 12 : 12 h light : dark cycle at 22°C and had free access to food but were water deprived throughout the tinnitus behavioural test. All procedures were approved by the University of Otago Committee on Ethics in the Care and Use of Laboratory Animals.

### 2.2. Acoustic Trauma to Induce Tinnitus

Unilateral acoustic trauma was delivered using a procedure described previously [35, 36]. Briefly, the animals were anaesthetised with fentanyl citrate (0.2 mg/kg, s.c.), medetomidine hydrochloride (Domitor, Novartis; 0.5 mg/kg, s.c.), and atropine sulphate (50 *μ*g/kg, s.c.) and placed inside a sound attenuation chamber. A 16 kHz 110 dB pure tone generated by a NI 4461 Dynamic Signal Acquisition and Generation system (National Instruments New Zealand Ltd.) was delivered to one of the ears for 1 h through a closed field magnetic speaker (Tucker-Davis Technologies) connected to a custom made speculum. Acoustic values were calibrated by connecting the speaker to a 0.25 inch prepolarised free-field microphone (Type 40BE, GRAS Sound & Vibration) via the speculum used to fit into the external auditory canal. The unexposed ear was blocked with cone-shaped foam and taped against the foam surface. The exposure was counterbalanced between the left and the right ears. The control animals were kept under anaesthesia for the same duration as the acoustic trauma animals, but without exposure.

### 2.3. Auditory Brainstem Responses

Auditory function in the exposed ears before and immediately after the acoustic trauma was measured using auditory brainstem-evoked response (ABR) thresholds described previously [37]. Briefly, the animals were anaesthetised as previously described and subdermal needle electrodes were placed at the vertex and over the bullae with a reference electrode at the occiput. Tone bursts of 5 ms duration (2 ms rise/decay, 1 ms plateau) were presented at a rate of 50/sec and in a series of decreasing intensity, which began at a level that resulted in distinct evoked potentials. Tone intensities progressed in 20-, 10-, and 5-dB steps, at 32, 20, 16, and 8 kHz, and the ABR threshold was determined as the lowest intensity that produced a visually distinct potential.

### 2.4. Cage Exchange to Induce Sleep Disturbance

Twenty-four hours after acoustic trauma, sleep disturbance was induced using the cage exchange method described by Cano et al. [32]. Briefly, rats in group 3 were placed in dirty cages previously occupied by another male rat for 1 week and left undisturbed for 5.5 h. The control and the acoustic trauma-only group rats were placed in clean cages for the same duration. The 5.5 h duration was chosen because insomnia and neuronal activation have been shown to be induced at this time point [32]. In order to confirm that cage exchange induced sleep disturbances and stress, the animal's behaviour inside the exchanged cage (dirty or clean cage) was recorded for 5.5 h and the sleep-wake cycle, that is, the latency to fall asleep, duration of sleep, and number of sleep episodes, was analysed [32].

### 2.5. Tinnitus Assessment

At 3 weeks after acoustic trauma, the animals were tested for the behavioural signs of tinnitus inside an operant conditioning test chamber (ENV-007, Med Associates Inc.) using the conditioned lick suppression method routinely used in our laboratory [37–40]. The animals were water deprived to 95% of their normal body weight and this ensured that the animals reliably produced approximately 2000 to 6000 licks per 15 min testing session. A broadband noise (BBN) was played throughout the 15 min session except at 10 random intervals, at which point 15 sec acoustic stimulus presentations were inserted. Two of the 10 presentations were always speaker- off periods (i.e, silence) and the remaining 8 were one of BBN, 20 kHz tones, or 32 kHz tones at one of 4 different intensity levels (BBN = 30 dB, 40 dB, 50 dB, 70 dB; 20 kHz = 70 dB, 80 dB, 90 dB, 100 dB; 32 kHz = 70 dB, 80 dB, 90 dB, 100 dB) in a random order with each stimulus repeated twice within each session. The type of stimulus varied randomly between sessions but remained constant within a session. The animals were trained to establish a conditioned lick suppression response by pairing the silence (conditioned stimulus) with mild foot shock (0.35 mA, 3 sec; unconditioned stimulus) produced by a constant current shock source (ENV-410B, Med Associates Inc.) through a scrambler (ENV-412, Med Associates Inc.). The magnitude of lick suppression was measured by comparing the number of licks in the period preceding the stimulus presentation (*A*) and the period during the stimulus presentation (*B*), that is, the suppression ratio (SR):

(1) $$\begin{array}{c} \text{SR}=\frac{B}{A+B}. \end{array}$$

Once the lick suppression was established (SR < 0.2), the rats were subjected to the frequency discrimination test, during which the acoustic stimuli were presented in the same way as in the acclimation and the suppression training. However, the foot shock was delivered only if the SR for the speaker off period was >0.2. If a rat did not have tinnitus, the presentation of the stimuli had no effect on its licking activity. However, if a rat had tinnitus, the tinnitus sound would serve as the conditioned stimulus instead of the silence. Therefore, a testing stimulus with similar acoustic features to its tinnitus should produce greater lick suppression in this rat than in control rats.

### 2.6. Immunohistochemistry

Four rats from the sham group and four rats that exhibited the behavioural signs of tinnitus were selected from each exposed-clean cage and exposed-dirty cage group. At the conclusion of the tinnitus behavioural testing, the rats were overdosed with sodium pentobarbital and perfused transcardially with 4% paraformaldehyde. The brains were dissected out, postfixed, and frozen. Forty *µ*m serial sagittal sections throughout the hypothalamus were collected according to a random, systematic sampling design for free floating immunolabelling. Antigen retrieval was achieved by incubating the sections in citrate buffer (pH 6) at 90°C for 10 min. The sections were then blocked with a blocking buffer (5% normal goat serum, 1% bovine serum albumin, and 5% Triton X-100 in 0.01 M PBS) for 2 h at room temperature before being incubated with a rat anti-orexin-A antibody (1 : 2000; Millipore, AB 3704) for 48 h at 4°C. This was followed by an incubation with a secondary antibody (horseradish peroxidase-conjugated goat anti-rabbit IgG, 1 : 200; Santa Cruz, sc-2004) for 2 h at room temperature. The orexin immunocomplex was visualised using a DAB kit. Every orexin-positive neuron was counted under a 63x oil objective lens throughout the thickness of the section in one set of the serial sections collected and the total number of orexin-positive neurons in the left and right hypothalamus was then estimated using a modified fractionator method [41–43].

### 2.7. Statistical Analysis

All data were tested for the normal parametric assumptions of normality and homogeneity of variance [44]. Where these assumptions were violated, the data were natural log or square root transformed and retested. Data were analysed using 2-way ANOVAs with repeated measures and multiple comparisons were conducted using Bonferroni tests. The number of animals exhibiting clear signs of tinnitus in each group was analysed using a chi-squared test. *P* ≤ 0.05 was considered significant.

## 3. Results

### 3.1. The Effect of Acoustic Trauma on Animals' ABR Thresholds

**Figure 1:**
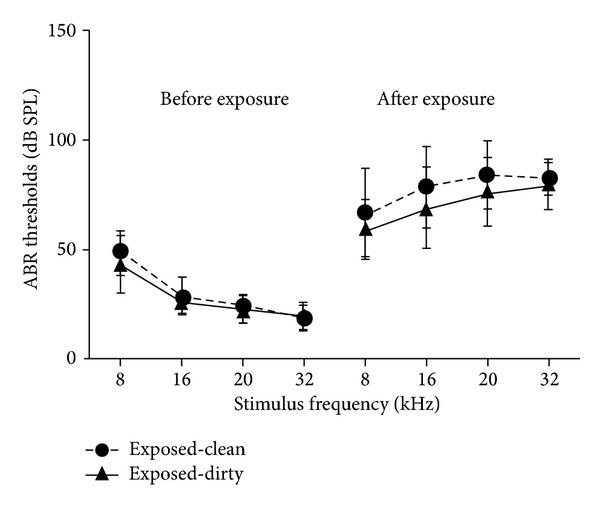
ABR thresholds at different stimulus frequencies in the ipsilateral ear of the animals from the exposed-clean and exposed-dirty cage groups measured pre- and postexposure to acoustic trauma. Symbols represent means ± 1 SEM.

Unilateral acoustic trauma caused an immediate increase in the ABR thresholds across all the frequencies examined (Figure 1). There was a significant exposure effect for both clean and dirty cage animals (*F*_3,175_ = 216.3, *P* ≤ 0.0001) and a significant frequency-exposure interaction (*F*_9,175_ = 10.76, *P* ≤ 0.0001), which suggests that the ABR threshold elevation was much greater at the higher frequencies compared with the lower frequencies.

### 3.2. The Effect of Cage Exchange on Animals' Sleeping Patterns

**Figure 2:**
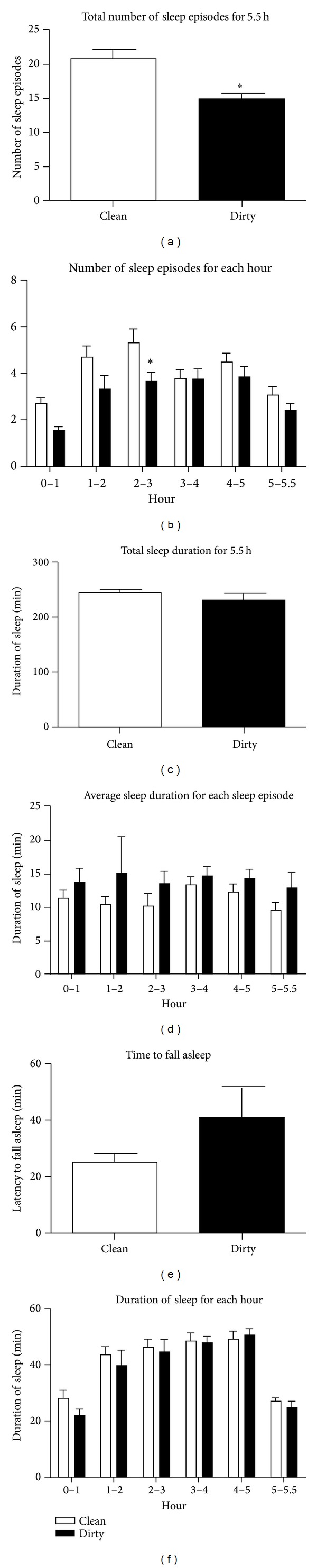
Sleep patterns during the 5.5 h cage exchange period. (a) Total number of sleep episodes during the 5.5 h period. (b) Number of sleep episodes during each hourly period. (c) Total duration of sleep during the 5.5 h period. (d) Average sleep duration for each sleep episode during each hourly period. (e) Time taken to fall asleep. (f) Sleep duration during each hourly period. Bars represent means ± 1 SEM.

The general behaviour of the rats placed in the dirty cages was not obviously different from those placed in the clean cages. However, after 5.5 h, the cage bedding was noticeably dishevelled, which suggests that the animals in the dirty cages were more unsettled. When the sleeping patterns were analysed, animals placed in the dirty cages had significantly fewer sleep episodes during the 5.5 h period when compared with those in the clean cages (*t* = 3.86, *P* ≤ 0.0012; Figure 2). A close inspection of the sleep episodes within each hourly period revealed that the reduced number of sleep episodes occurred mainly during the first 3 h inside the dirty cages, with the significant reduction being observed between 2 and 3 h (*F*_1,17_ = 14.86, *P* ≤ 0.0013; Figure 2). There was no difference between the clean and dirty cage groups in the time taken to fall asleep and the duration of sleep, whether it was measured during the 5.5 h or within each hourly period (Figure 2).

### 3.3. The Effects of Cage Exchange on Animals' Tinnitus Perception

**Figure 3:**
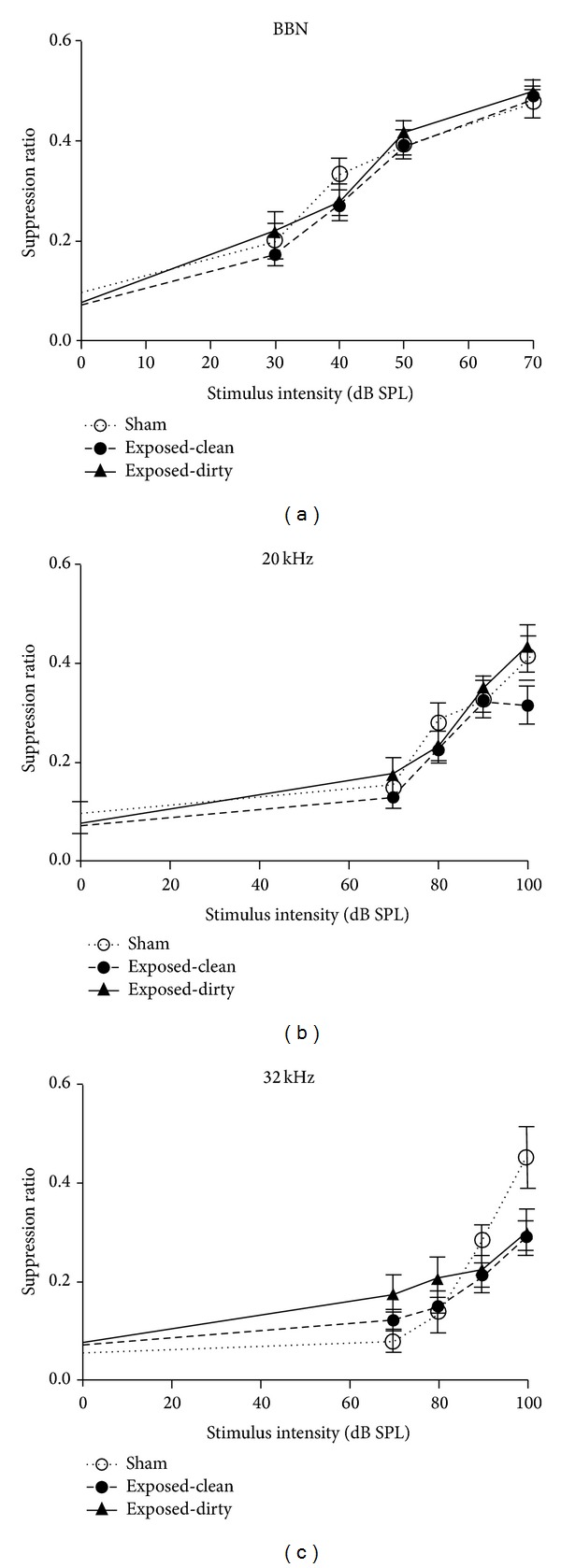
Frequency discrimination curves for suppression ratio in sham, exposed-clean, and exposed-dirty animals in response to BBN, 20 kHz or 32 kHz stimuli at different intensities. Symbols represent means ± 1 SEM.

Frequency discrimination curves for all of the groups were plotted using the SRs against the intensity of each frequency of the stimulus (Figure 3). As expected, SR values differed significantly with different stimulus intensities for BBN (*F*_4,108_ = 134.7, *P* ≤ 0.0001), 20 kHz (*F*_4,108_ = 64.42, *P* ≤ 0.0001), and 32 kHz (*F*_4,108_ = 43.08, *P* ≤ 0.0001) stimuli. While there was no significant group difference when tested for any of the 3 stimuli presentations, there was a significant intensity *x* group interaction specifically for the 32 kHz tones (*F*_8,108_ = 2.954, *P* ≤ 0.005). Multiple comparisons revealed that 32 kHz tones elicited a significant downward shift of the curves at 100 dB for both the exposed-clean and exposed-dirty groups compared with the sham group (*t* = 3.071, *P* ≤ 0.05, for the exposed-clean group; *t* = 2.950, *P* ≤ 0.05, for the exposed-dirty group). The number of animals exhibiting clear behavioural signs of tinnitus was not significantly different between the exposed-clean (4 out 12) and exposed-dirty (5 out of 12) groups (*P* = 1.000, chi-square test).

### 3.4. The Effects of Cage Exchange and Tinnitus on the Number of Orexin Neurons in the Hypothalamus

**Figure 4:**
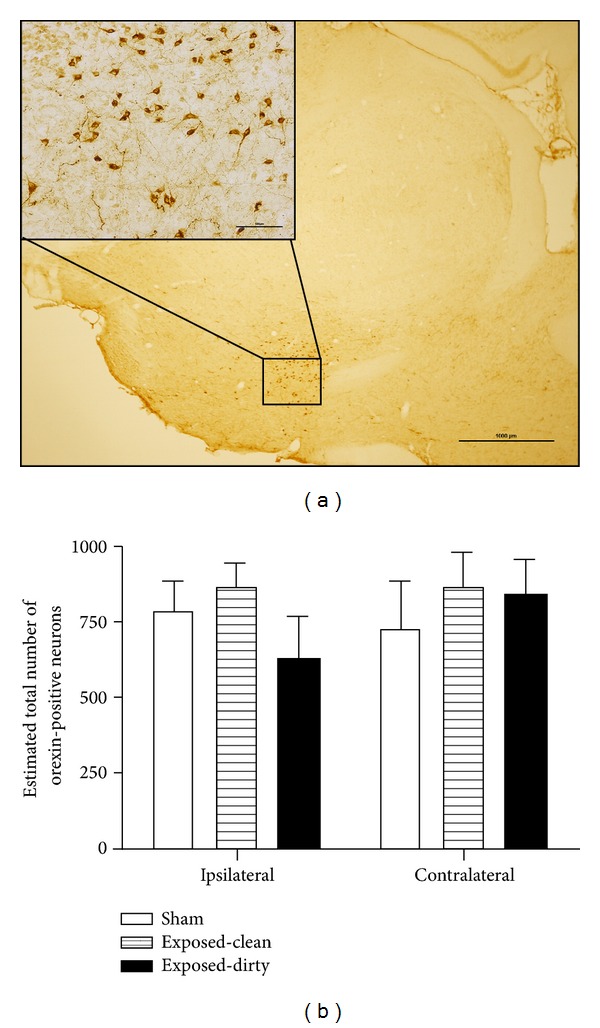
Orexin immunoreactivity in the hypothalamus. (a) A photograph showing orexin immunostaining located in the hypothalamus under a low magnification (scale bar: 1000 *µ*m). Inserted picture showing the presence of orexin-positive staining in the cytoplasm and the processes of the neurons (scale bar: 100 *µ*m). (b) Estimated total number of orexin-positive neurons in the ipsilateral and contralateral hypothalamus in sham, exposed-clean, and exposed-dirty animals. Bars represent means ± 1 SEM.

Orexin immunohistochemistry was carried out in four nontinnitus rats from the sham group, four tinnitus rats from the exposed-clean group, and four tinnitus rats from the exposed-dirty group and revealed selective and specific staining in the cytoplasm of the cell bodies and the processes of neurons throughout the hypothalamus (Figure 4(a)). Orexin-positive fibres were present in many areas of the brain including the cochlear nucleus; however, no orexin-positive cell bodies were observed in any areas of the brain other than the hypothalamus (data not shown). Stereological cell counting showed that there was no significant difference in the total number of orexin-positive neurons in the hypothalamus either between the ipsilateral and contralateral sides to the acoustic trauma-exposed ear or among the sham, exposed-clean, and exposed-dirty groups (Figure 4(b)), which suggests that neither tinnitus alone nor tinnitus plus sleep disturbance altered the number of orexin-expressing neurons.

## 4. Discussion

Our results showed that stress-inducing cage exchange caused a sleep disturbance and acoustic trauma-induced tinnitus in rats. However, sleep disturbance did not affect tinnitus perception caused by acoustic trauma. Furthermore, sleep disturbance and tinnitus did not change the number of orexin neurons in the hypothalamus.

The cage exchange method to induce sleep disturbance is based on the notion that stress is the most common cause of sleep disorders. This method, by placing the rat in a dirty cage previously occupied by another male rat for 1 week, has been shown to successfully induce sleep disturbances in rats with similar behavioural and electrophysiological patterns to those observed in stress-induced insomnia in humans [32]. In addition, using c-Fos as an indication of neuronal activation, the study also showed an activation of the brain areas involved in arousal and emotional control. It was hypothesized that “being inescapably surrounded by the territory that has been marked by another male rat” is the psychological stressor rather than the odour of another rat itself, since the animals were housed next to each other [32]. Indeed, compared with the rats being placed in clean cages, the rats in the dirty cages were noticeably unsettled, which indicated an increase in stress. Analysis of the sleeping patterns revealed that animals in dirty cages had significantly fewer sleep episodes compared with the animals in clean cages, albeit with no difference in sleep durations. The decrease in the number of sleep episodes was observed during the third hour after cage exchange, which is similar to what was reported previously [32]. However, the dirty cage animals in our study had similar latencies to fall asleep and slept for a similar duration as the clean cage animals. These findings are in contrast with the previous study where a longer latency to fall asleep and an increased wakefulness were reported [32]. One of the factors that might contribute to the differences between the studies is that our rats received anaesthesia 24 h prior to the cage exchange and the anaesthesia might have affected the level of stress induced by cage exchange. Although it is difficult to determine whether the duration of sleep or the number of sleep episodes is more important for the quality of sleep in rats, the fact that sleep in rats was highly fragmented suggests that the reduced sleep episodes in dirty cage animals may reflect a reduced quality of sleep. However, changes in sleep quality did not exacerbate the behavioural signs of tinnitus in these animals.

Tinnitus was induced by exposing the rats to a 16 kHz pure tone at 110 dB SPL for 1 h. Acoustic stimuli at the intensities between 110 and 115 dB SPL have successfully induced tinnitus in rats in our previous studies [37–40, 45]. We chose to use the intensity at the lower end, that is, 110 dB SPL, for this study with the intention of causing a moderate level of tinnitus in the exposed-clean group and to increase the sensitivity to detect a difference between animals in the clean and dirty cages if the dirty cages made tinnitus worse. As expected, this acoustic trauma parameter induced clear behavioural signs of tinnitus in 33% of the animals (4 out of 12 animals) in the clean cage group, which is lower than our previous tinnitus induction rate (62.5–75%) when a 115 dB tone was used. However, dirty cages did not significantly increase the number of animals exhibiting tinnitus (5 out of 12 animals). We found acoustic trauma-induced tinnitus with acoustic features similar to a 32 kHz tone of 100 dB in both the clean and dirty cage animals and there was no significant difference between the clean and dirty cage animals. Given that acoustic trauma caused comparable elevations of the ABR thresholds immediately after the exposure in both groups of animals, the peripheral damage caused by acoustic trauma should also be similar between the two groups. Therefore, additional stress and sleep disturbance 24 h after acoustic trauma did not increase tinnitus severity in this experiment. If a stress factor was introduced during or immediately after the acoustic trauma, it might be possible to increase tinnitus severity. However, under the current animal ethics regulation, that is, the acoustic trauma was delivered under anaesthesia, this possibility could not be explored. Another reason that sleep disturbance failed to intensify tinnitus might be that the stress-inducing cage exchange was delivered only once following acoustic trauma and the sleep disturbance did not last long enough. Methods to induce chronic stress or chronic sleep disturbance may be needed in order to further investigate the relationship between stress and tinnitus perception.

Since hyperarousal has been proposed to underlie both tinnitus and insomnia (see [19] for a review) and the orexin system plays an important role in maintaining long-lasting arousal (see [27] for a review), it remains unknown whether the orexin system could be altered in animals that developed tinnitus and/or sleep disturbance. In this study, the total number of orexin-expressing neurons was estimated using immunohistochemistry and stereology throughout the hypothalamus. No significant difference was found between the different groups. Different types of stimulations, such as insulin-induced hypoglycemia, caffeine, and stress, have been shown to activate orexin neurons in the hypothalamus evaluated by measuring c-Fos expression in orexin neurons [46]. It is possible that changes in the orexin system are mediated by activation of the existing orexin neurons rather than the induction of new orexin neurons. Another possibility is that there is an increased orexin projection to the target areas, since orexin neurons project to a wide range of brain areas including the inferior colliculus and the ventral cochlear nucleus [26]. Therefore, an increase in orexin neurotransmission in these auditory brain areas might be related to tinnitus. Further studies are needed to examine these possibilities following acoustic trauma-induced tinnitus.
